# Acute and Chronic Management of Ocular Disease in Stevens Johnson Syndrome/Toxic Epidermal Necrolysis in the USA

**DOI:** 10.3389/fmed.2021.662897

**Published:** 2021-07-12

**Authors:** Derek Metcalfe, Omer Iqbal, James Chodosh, Charles S. Bouchard, Hajirah N. Saeed

**Affiliations:** ^1^Massachusetts Eye & Ear Infirmary, Harvard Medical School, Boston, MA, United States; ^2^Loyola University Medical Center, Maywood, IL, United States

**Keywords:** Stevens-Johnson syndrome, toxic epidermal necrolysis, keratoprosthesis, amniotic membrane transplantation, ocular SJS, mucous membrane graft

## Abstract

Stevens Johnson syndrome and toxic epidermal necrolysis are on a spectrum of a severe, immune-mediated, mucocutaneous disease. Ocular involvement occurs in the vast majority of cases and severe involvement can lead to corneal blindness. Treatment in the acute phase is imperative in mitigating the severity of chronic disease. Advances in acute treatment such as amniotic membrane transplantation have shown to significantly reduce the severity of chronic disease. However, AMT is not a panacea and severe chronic ocular disease can and does still occur even with aggressive acute treatment. Management of chronic disease is equally critical as timely intervention can prevent worsening of disease and preserve vision. This mini-review describes the acute and chronic findings in SJS/TEN and discusses medical and surgical management strategies.

## Introduction

### Overview

Stevens-Johnson syndrome (SJS) and toxic epidermal necrolysis (TEN) are severe and potentially lethal multisystem, mucocutaneous, immune-mediated, adverse drug reactions (IM-ADR), with significant long-term ocular and systemic morbidity (1–6). Medications trigger SJS/TEN in >80% of adults, typically occurs within the first few weeks after first administration or upon a dose adjustment of an inciting agent (culprit drug) (7). Secondary complications include sepsis, blindness, respiratory, and genitourinary scarring and dysfunction.

Severe cicatrizing ocular surface disease is one of the most significant and debilitating sequelae of SJS/TEN and can profoundly impact the patient's quality of life (QOL) (8, 9). There is a short window of opportunity during the acute stage where intervention may potentially avoid these lifelong complications including severe vision loss and blindness.

### Etiology and Culprit Drugs

Although the induction of SJS/TEN may be multifactorial, medications are the most common culprit (10–12). A large study of 377 patients in the US between 2000 and 2015 found that antibiotic agents were the most common class of culprit drug with trimethoprim-sulfamethoxazole in 26.3% of cases (13). Anti-epileptics, particularly carbamazepine, lamotrigine, and phenytoin as well as allopurinol are other common causes of SJS/TEN (10, 14, 15).

## Treatment Strategy for Sjs/Ten With Severe Ocular Complications in the Acute Stage (0–6Weeks)

### Presenting Signs and Symptoms

The clinical presentation often begins with a prodrome of fever, malaise, cough, rhinorrhea, and anorexia followed by mucositis and a painful generalized erythematous vesiculobullous rash with skin sloughing (2, 5, 16). Early ocular disease is highly variable and can range from conjunctival hyperemia, sometimes as early as the prodromal phase, to near total sloughing of the entire ocular surface and eyelid margin epithelium.

### Initial Eye Exam

An initial ocular examination on *all SJS/TEN patients* should occur within 24 h of admission. However, only 66% of burn ICUs in the United States consult ophthalmology for SJS/TEN patients (17, 18). A standardized EMR template may be useful to facilitate and prompt the documentation of key clinical signs on a daily basis. Examination should include the upper and lower eyelid skin, eyelid margins, and meibomian gland orifices. Fluorescein dye should be used to assess epithelial breakdown of the eyelid margins and ocular surface (cornea and conjunctiva). The entire conjunctiva including the forniceal and tarsal conjunctiva should be examined by everting the eyelids, with special attention to the presence of membranes (5, 19, 20). Saline rinses can aid in removal of mucous and tear film debris that may hide corneal or conjunctival epithelial defects (18).

### Subsequent Examinations

Following initial ocular examination, patients should be monitored every 24–48 h during the first week of admission due to the potential for clinical signs and symptoms to rapidly progress (5). Daily evaluation is needed for any patient with the following: eyelid margin involvement, conjunctival pseudomembranes, opposing bulbar and tarsal conjunctival defects, or corneal epithelial defects. Upper and lower fornices need to be inspected daily. Degree of eyelid margin staining (location, size) should also be documented. The length and width of any corneal epithelial defect(s) should be measured and recorded.

**Figure 1:**
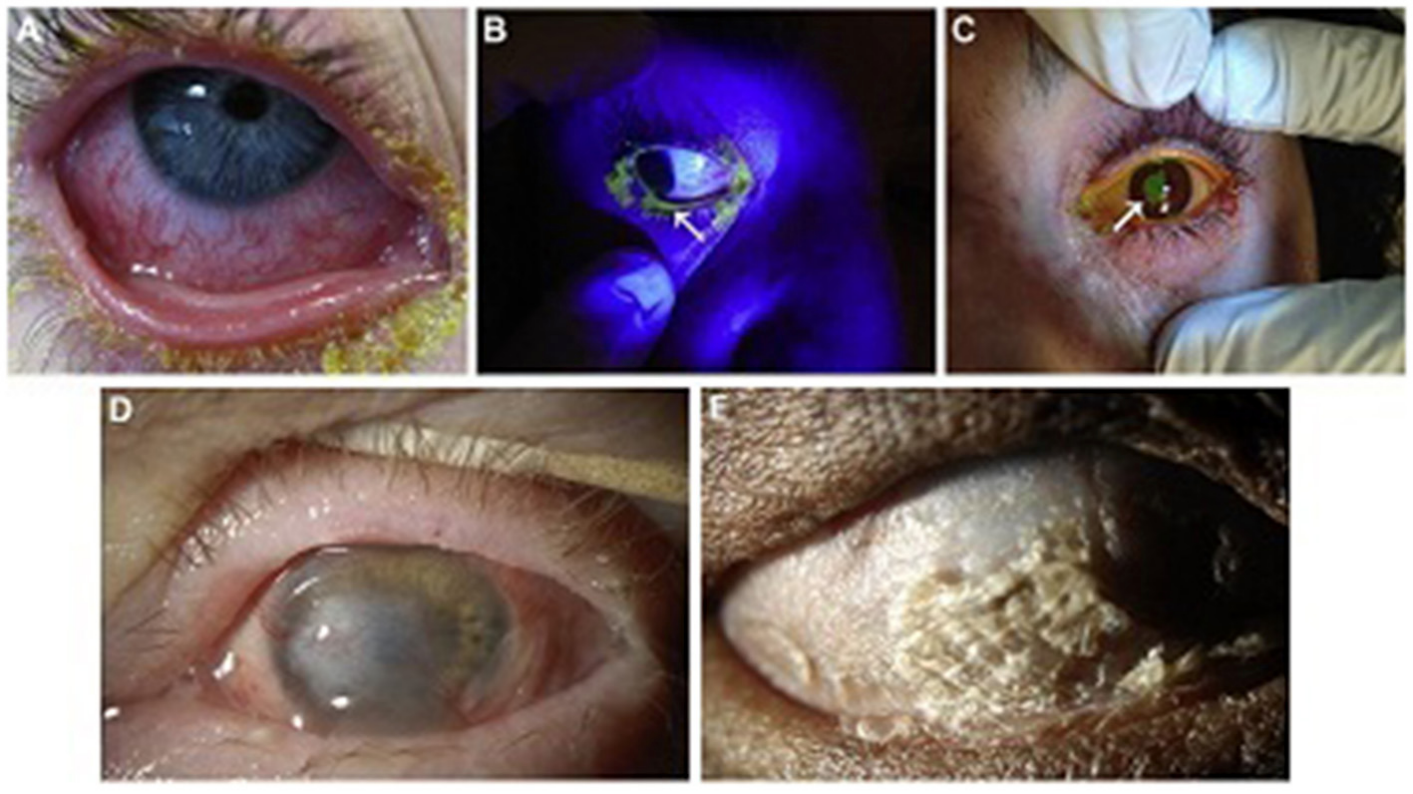
Ocular surface involvement in acute SJS/TEN and severe chronic SJS/TEN. **(A)** Acute conjunctival hyperemia and membrane. **(B)** Acute eyelid margin sloughing (arrow) as evident with fluorescein staining under cobalt blue light. **(C)** Acute corneal epithelial defect (arrow) stained with fluorescein. **(D)** Chronic dense corneal neovascularization and opacity in a wet, blinking eye. This eye might be a candidate for a Boston keratoprosthesis type I. **(E)** Complete ocular surface keratinization in an eye devoid of aqueous tears. This eye would not be a candidate for a Boston keratoprosthesis type I, but might be for a Boston keratoprosthesis type II. Reproduced with permission from Elsevier (19).

The position of the eyelid should also be noted, as lagophthalmos, either from intubation/sedation or early cicatricial changes, can lead to corneal exposure with blinding complications (5, 19). A Desmarres retractor is useful in facilitating the examination and rotating the upper eyelid. See Figures 1A–C for examples of ocular involvement in acute SJS/TEN.

## Management Protocols for Acute Ocular Sjs/Ten

### Ocular Surface Disease Severity Grading and Treatment Overview

Several grading systems have been developed to assess disease severity in the acute stage. Sotozono et al. developed a grading system in 2007 to classify the severity of acute ocular disease (8). Their grading scheme lacked eyelid margin involvement, which has become an important risk factor for chronic surface disease (21). An updated grading system and algorithm for the initiation of ocular therapy in SJS/TEN is presented in Supplementary Figure 1 (5, 22, 23). Beneficial long-term outcomes following the use of this standardized protocol has been recently published (17, 23).

Suppression of exogenous and endogenous causes of inflammation, avoidance of treatment toxicity, and preservation of the ocular surface are essential to halt disease progression (1, 5, 19). Resolution of conjunctival injection, epithelial defects, and eyelid margin ulceration are signs of resolution of acute disease.

### Medical Management

Ocular treatment should start on admission as it is critical to maximally inhibit the ocular immune response to minimize long-term scarring. Ocular disease may actually precede the severe skin changes and treatment should not be delayed for skin biopsy results. Coordinated effort with burn unit/ICU nursing staff with written protocols is essential.

For Grades 0–1, daily saline (NaCl 0.9%) flushes should be performed and pseudomembranes debrided with a cotton tipped applicator. Medical treatment should include moxifloxacin 0.5% drops three times a day, topical steroid eye drop six times a day, and a steroid or antibiotic-steroid combination ointment to the eyelid margins 4–6 times a day. Cyclosporine 0.09% drops (Cequa, Sun Pharma) four times a day should also be considered. Preservative-free artificial tears should be used every 1–3 h in between the other drops.

In addition to topical antibiotics, small corneal epithelial defects may be managed initially with lubrication and/or soft therapeutic contact lenses to aid healing and minimize trauma. Larger defects may require amniotic membrane (ProKera) (see below).

All cases of cicatricial lagophthalmos and associated exposure should be aggressively managed with frequent lubricant ointment, humidity goggles, and/or plastic wrap to address evaporative dry eye. Definitive management includes surgical release of the cicatrix. Sedation-induced lagophthalmos (non-cicatricial) can be effectively managed with Tegaderm (3M, Saint Paul, MN) placement (19). For prevention of exposure keratopathy, scleral contact lenses have also been effective (19, 24, 25). External photography is very helpful to follow disease if available.

For Grades 2–3 with significant eyelid margin involvement +/– bulbar conjunctiva, amniotic membrane should be applied in addition to the above medical management (see below).

### Surgical Management: Amniotic Membrane Transplantation (AMT)

All patients with eyelid margin involvement, pseudomembranes, and/or corneal and conjunctival epithelial defects within 4–7 days from index day, should receive AM (19, 23, 26, 27). Better visual acuity and reduced incidence of corneal haze, limbal stem cell deficiency (LSCD), symblepharon, ankyloblepharon, or eyelid-related complications have been reported in the long-term (17, 28). Although AM has not been shown to affect mild dry eye, it has been shown to affect the incidence of moderate to severe dry eye (17).

Timing for AM placement is crucial, as its anti-inflammatory and anti-scarring properties are more beneficial when used early on in the acute phase. Previous studies have reported timeframes between 5 and 10 days after symptom onset as the ideal window for prevention of serious long-term ocular complications (20, 23). AM can dissolve anywhere from 3 days to 2 weeks post placement (5, 19) and can be repeated as necessary in patients with persistent inflammation. Complications are exceedingly rare (17, 29–32).

AM can be used either as a large single sheet of AM or in the form of a ProKera device (Biotissue, Miami, FL), but a ProKera by itself is insufficient. Prokera is an AM that is stretched across a polycarbonate ring and is placed on the eye similar to a contact lens (26). Although it can be inserted at the bedside without sedation and quickly replaced, it does not cover peripheral conjunctiva, fornices, and eyelid margins and leaves these areas susceptible to complications (26, 27, 33). Single sheet AMT utilizes a single 5 × 10 cm sheet of AM that is secured to the upper and lower eyelids, by suture or glue, and a large symblepharon ring that is inserted to ensure contact of the membrane to the upper and lower fornix. A recent study by Shanbhag et al. describes a sutureless AMT technique involving cyanoacrylate glue to secure the AM to the eyelids (34). It has been shown to speed up AMT placement as well as cause less discomfort allowing for bedside treatment without general anesthesia.

If a patient is already scheduled for a procedure in the OR, ophthalmologists should consider placement of AMT within the same scheduled time. If bedside or operative room AMT placement are not possible due to instability of the patient's condition, patient declining treatment, or due to issues of comfort/patient cooperativity, ProKera can be placed but it will not prevent later eyelid margin keratinization. It is important to note that AMT is also not a panacea and severe chronic disease can still occur despite aggressive acute phase treatment and all SJS/TEN patients must be followed closely for the development of complications (17, 23).

### Systemic Treatment

Systemic treatment may provide benefit in managing the ocular disease in acute SJS/TEN. Suggested therapies as reported include the use of corticosteroids, intravenous immunoglobulin (IVIG), plasmapheresis, cyclosporine, granulocyte-stimulating factor, TNF-alpha inhibitors, and cyclophosphamide but published results are equivocal at best (5, 19, 35–40). In addition, these treatments are not without systemic risks. An FDA approved prospective randomized clinical trial (NATIENS) is planned in the US comparing etanercept, cyclosporine and supportive therapy (https://clinicaltrials.gov/ct2/show/NCT02987257?term=natiens&draw=2&rank=1).

## Treatment Strategy for Sjs/Ten With SOC in the Chronic Stage

### Overview of Management Strategy for Chronic Disease

Thirty-50% of patients who survive SJS/TEN in the acute setting develop some form of chronic ocular disease and all patients should have a follow-up ophthalmologic exam (41). The initial follow up visit should be performed within the first month after discharge and should be repeated every 2–4 months in the first year and then at least every 6 months thereafter, dependent upon the patient's ocular condition (19). Chronic complications for which these acute management strategies are designed to prevent and treat include those related to dry eye disease (DED), eyelid malposition (ectropion, entropion, lagophthalmos), misdirected lashes (trichiasis, distichiasis), posterior eyelid margin keratinization, conjunctival disease (keratinization, symblephara, ankyloblephara), and corneal disease (epithelial defects, thinning, scarring, neovascularization, LSCD). There is no standardized timepoint at which SJS/TEN is considered to be chronic, but in general this is related to stabilization of any acute inflammation and de-epithelialization. This may be between the 3–6 months post-SJS-onset range.

### Ocular Surface Stabilization

Ocular surface inflammation can persist and/or recur episodically during the chronic phase (42). Failure to stabilize the ocular surface can result in postoperative inflammatory and infectious complications (43, 44). Topical antibiotics and low potency topical corticosteroids may be needed for treatment of brief bouts of inflammation. High potency topical corticosteroids can be associated with infection and/or keratolysis and long-term use is ill-advised (19). Oral doxycycline or azithromycin may help in controlling inflammation (45). Systemic immunosuppressive therapy with cyclosporine, azathioprine and others may have a role in stabilization, although studies were performed without controls (42, 46).

Ocular surface inflammation may be due in part to changes in the ocular surface microbiome (44, 47–49). SJS/TEN eyes may have a more diverse microbiome than healthy eyes and may be due to deficiencies in the innate immune response (50). This imbalance may result in inflammation leading to ulceration and infection (51–53). These results raise interesting possibilities for clinical management of the disease including probiotics to promote the growth of a healthy microbiome or targeted antibiotics to kill pathogenic bacteria that may be causing inflammatory symptoms. Further investigation is warranted to better understand the immunopathophysiology and potential targets for intervention.

### Ocular Xerosis and Dry Eye Disease (DED)

DED is a common complication of SJS/TEN, occurring in more than 50% of patients secondary to deficiencies in all three components of tear film: aqueous, mucin, and lipid (19, 54–56). Topical cyclosporine has had equivocal success in improving goblet cell density, possibly due in part to self-withdrawal due to side effects (57). For aqueous deficiency, preservative-free artificial tears may be used but require frequent dosing and can be expensive. Punctal occlusion (cautery or plugs) can improve ocular surface health; many patients may already have closed puncta from scarring related to SJS/TEN (58). Minor salivary gland transplantation can increase ocular surface wetting and corneal clarity (3, 19, 59–61). Serum tears have also been reported to improve clinical signs and symptoms (3, 19, 62).

### Abnormalities of the Eyelid and Lashes

Malposition of the eyelids and misdirection of the eyelashes is a common chronic sequela of SJS/TEN. Trichiasis and distichiasis can be temporarily treated with mechanical epilation, whereas long-term treatment involves hyfrecation, cryotherapy, and/or extirpation (19). Ectropion or entropion can be treated with lateral canthoplasty or tarsal strip, anterior lamellar repositioning, tarsal fracture, posterior lamellar tightening, or tarsoconjunctival advancement (19). Tarsorrhaphy and cicatricial release can be used to treat lagophthalmos as well. In the setting of posterior eyelid margin keratinization or scarring resulting in entropion, mucous membrane grafting (MMG) or scleral lenses such as the PROSE lens are most appropriate (5, 63, 64).

### Primary and Secondary Corneal Complications

Corneal infection and perforation are severe consequences that can occur as a result of persistent, untreated corneal epithelial defects during the acute and subacute phase of SJS/TEN. Recommended standard treatments for persistent defects include those that modulate tear film (lubrication with artificial tears and ointment, serum tears, punctal occlusion), those that protect the ocular surface [discontinuation of toxic medications, bandage contact lens, AMT (ProKera)], and those that correct eyelid abnormalities (tarsorrhaphy) (19, 62, 65–67).

Posterior eyelid margin keratinization is itself a primary cause of corneal disease from repetitive mechanical microtrauma which can induce corneal epithelial defects, infection, perforation, and stromal melting as well as LSCD and ultimately corneal blindness (21, 68, 69). Treatment for posterior eyelid margin keratinization includes all-trans retinoic acid ointment (70, 71), scleral contact lenses such as PROSE therapy (63, 72) and MMG (41, 60, 73).

PROSE is a treatment that utilizes a gas-permeable, large-diameter contact lens which provides a protective barrier over the cornea and submerges the entire corneal surface in a pool of oxygenated artificial tears creating an environment which supports healing and maintenance of the corneal epithelium (63, 74). It also improves visual acuity and comfort and reduces corneal complications (41, 64, 75). Symblephara may need management before a lens can be fitted properly (21). Newly developed limbal rigid contact lenses may be indicated in eyes with a short fornix and/or symblepharon (76, 77).

Definitive treatment for posterior eyelid margin keratinization is MMG (41, 60, 62). By replacing the keratinized mucosal surface with healthy, viable mucosa, typically from the oral cavity, the procedure removes the microtrauma associated with a keratinized eyelid. MMG has been shown to restore ocular surface integrity and improve visual function, particularly when used in conjunction with PROSE devices, in both children and adults (41, 78, 79). MMG can be performed in conjunction with autologous cultivated oral mucosal epithelial transplantation (COMET), a technique that utilizes host oral mucosa as a graft and transplants it onto the corneal surface (80, 81). Allogeneic simple limbal epithelial transplantation (SLET) may also be used in conjunction with MMG to address LSCD in eyes without extensive cicatrization and with a wet surface (82).

## Management Protocols for End-Stage Therapy

### Globe Salvage and Ocular Surface Stabilization

Ocular surface keratinization from SJS/TEN can actually be protective of the ocular surface but at the cost of severely reduced vision. Once disease has progressed to this stage, it is unlikely that ocular surface reconstructive surgery alone will restore visual function. See Figures 1D,E. However, there may be a short window prior to this point at which some globe salvaging therapy remains viable. This includes scleral contact lenses for non-healing epithelial defects, cyanoacrylate glue with a bandage contact lens for small perforations or keratolysis, and Gunderson conjunctival flap (19). Penetrating keratoplasty may be utilized for severe corneal thinning/perforation or corneal infection with thinning but leaves patients at further risk for complications such as graft ulceration and perforation and reactive inflammatory response (19).

Ocular surface stabilization procedures should be considered in order to restore normal eyelid/globe anatomy and improve tear film before consideration of reconstruction. These include punctal occlusion to improve tear film, MMG for posterior eyelid margin keratinization, and AMT with/without MMG, or COMET to restore conjunctival fornices (19, 70). Most reports on limbal stem cell transplantation in the setting of SJS/TEN, either show poor outcomes or are series with limited follow up. Short-term improvement in vision and the ocular surface is most often not sustained in the long-term (83). Allogeneic SLET may be an option in select eyes with LSCD and may have better outcomes compared to other forms of limbal stem cell transplantation in this population (82).

### Reconstructive Management

**Figure 2:**
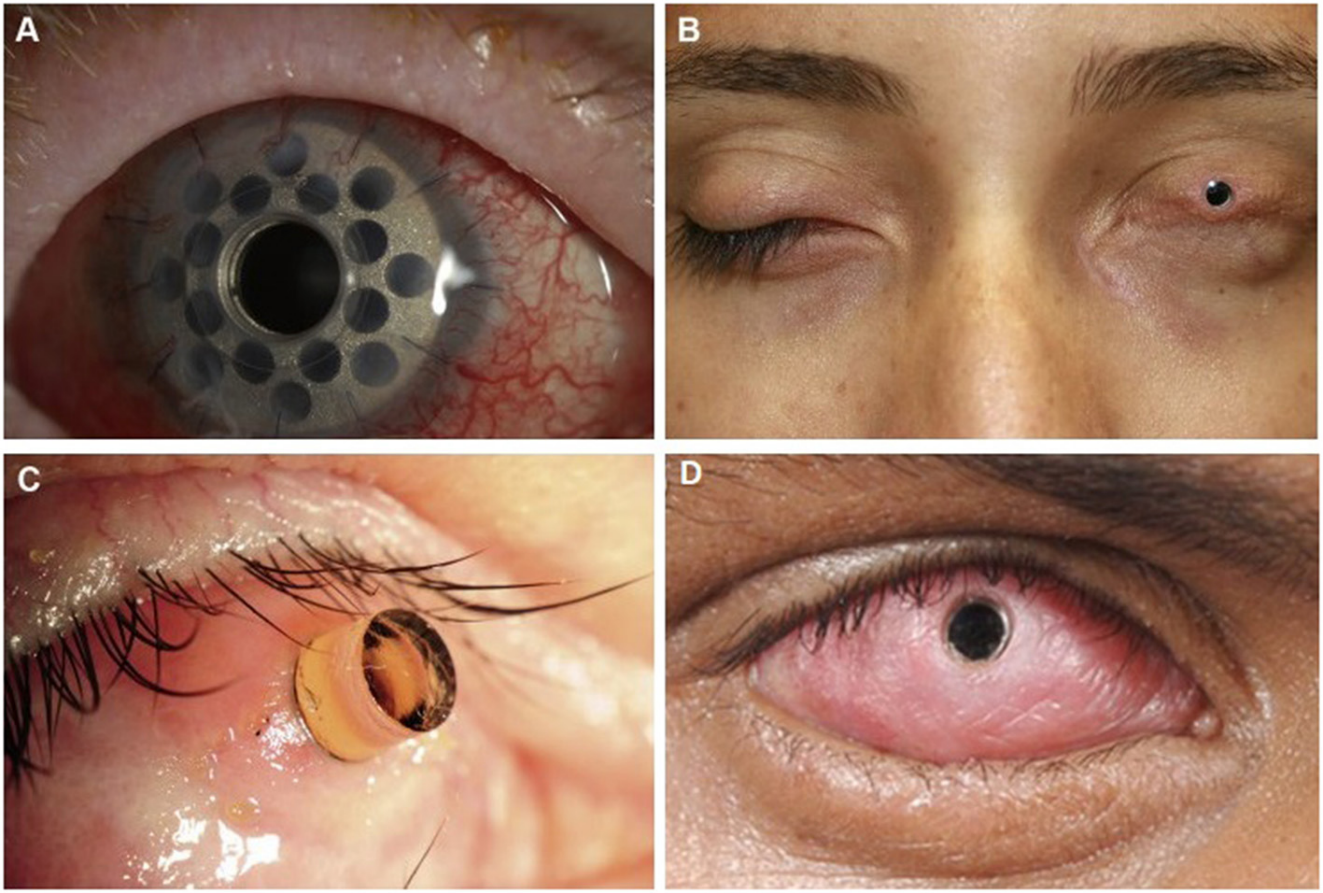
Keratoprosthesis implantation in patients post SJS/TEN. **(A)** Boston keratoprosthesis type I. **(B)** Boston keratoprosthesis type II. **(C)** Osteo-odonto-keratoprosthesis. This image is taken from an oblique view. **(D)** LVP Keratoprosthesis. Reproduced with permission from Elsevier and BMJ Publishing Group Ltd. (19, 109).

Keratoprosthesis (KPro) is the mainstay of visual rehabilitation in end-stage ocular SJS/TEN as it has been shown to restore normal to near normal visual function after surgery, although not indefinitely as complications and the need for repeat procedures often arise (41, 84–91). Unfortunately, relative to other populations, patients with SJS/TEN tend to have worse post-operative complications, device retention, and visual prognosis after KPro; KPro should be considered an option of last resort (92–95). Common complications include melt and leaks, endophthalmitis, microbial keratitis, and glaucoma (96–102). The different types of keratoprostheses include Boston KPro (types I and II), the LVP KPro, and the modified osteo-odonto-keratoprosthesis (MOOKP). Boston KPro type I can only be done in eyes with a wet ocular surface and intact eyelid function. The Boston KPro type II, MOOKP, and LVP KPro may be done in eyes with a dry, keratinized surface and with significant eyelid abnormalities (103–109) (see Figure 2).

## Conclusions and Future Direction

SJS/TEN is a severe multisystem, immune-mediated mucocutaneous disease commonly involving the ocular surface that has the potential to result in corneal blindness. The ophthalmologist is a critical caretaker in the acute and long-term treatment of these patients. Early aggressive intervention using a standardized protocol as that proposed is vital to reduce and/or prevent chronic ocular morbidity. As chronic disease may still arise regardless of early treatment, interventions such as PROSE and MMG in the chronic phase, and keratoprosthesis at end-stage disease, may be necessary. Prevention of significant disease should be the mainstay of future research and includes more targeted acute ocular and systemic therapy; identification of biomarkers for early diagnosis of disease and for prognostic assessment; and education and training of healthcare personnel on early referral to tertiary burn care centers, standardized treatment protocols, and windows of treatment opportunity. To truly mitigate the occurrence of ocular surface and systemic disease from SJS/TEN, personalized medicine in the form of genetic screening is needed to identify at-risk individuals and prevent rather than treat the occurrence of disease.

## Author Contributions

DM, OI, CB, and HS contributed to conception and design of this review. DM did the literature review and organized the structure of the review. DM, CB, and HS wrote the first draft of the manuscript. OI and JC wrote sections of the manuscript. All authors contributed to manuscript revision, read, and approved the submitted version.

## Conflict of Interest

HS and JC are employed by Mass Eye and Ear which manufactures and distributes the Boston keratoprosthesis and is discussed in this article. The remaining authors declare that the research was conducted in the absence of any commercial or financial relationships that could be construed as a potential conflict of interest.

## Supplementary Material

Supplementary Figure 1 Flow diagram outlining the protocol for management of ocular manifestations in acute SJS/TEN. MF [moxifloxacin 0.5%; PA [prednisolone acetate 1%; FML [fluorometholone 0.1%; AT [artificial tears; AMT [amniotic membrane transplantation. ^*^Decision to perform AMT was based on feasibility (intubation status, cooperation, etc.). ProKera is acceptable only with limited bulbar conjunctival or corneal involvement or when AMT is not feasible. Reproduced with permission from Elsevier (23).
