# Supplementary figures and images for: Acute and Chronic Management of Ocular Disease in Stevens Johnson Syndrome/Toxic Epidermal Necrolysis in the USA
Source: Front Med (Lausanne). 2021 Jul 12;8:662897. doi: 10.3389/fmed.2021.662897 (PMC8311126; doi:10.3389/fmed.2021.662897)

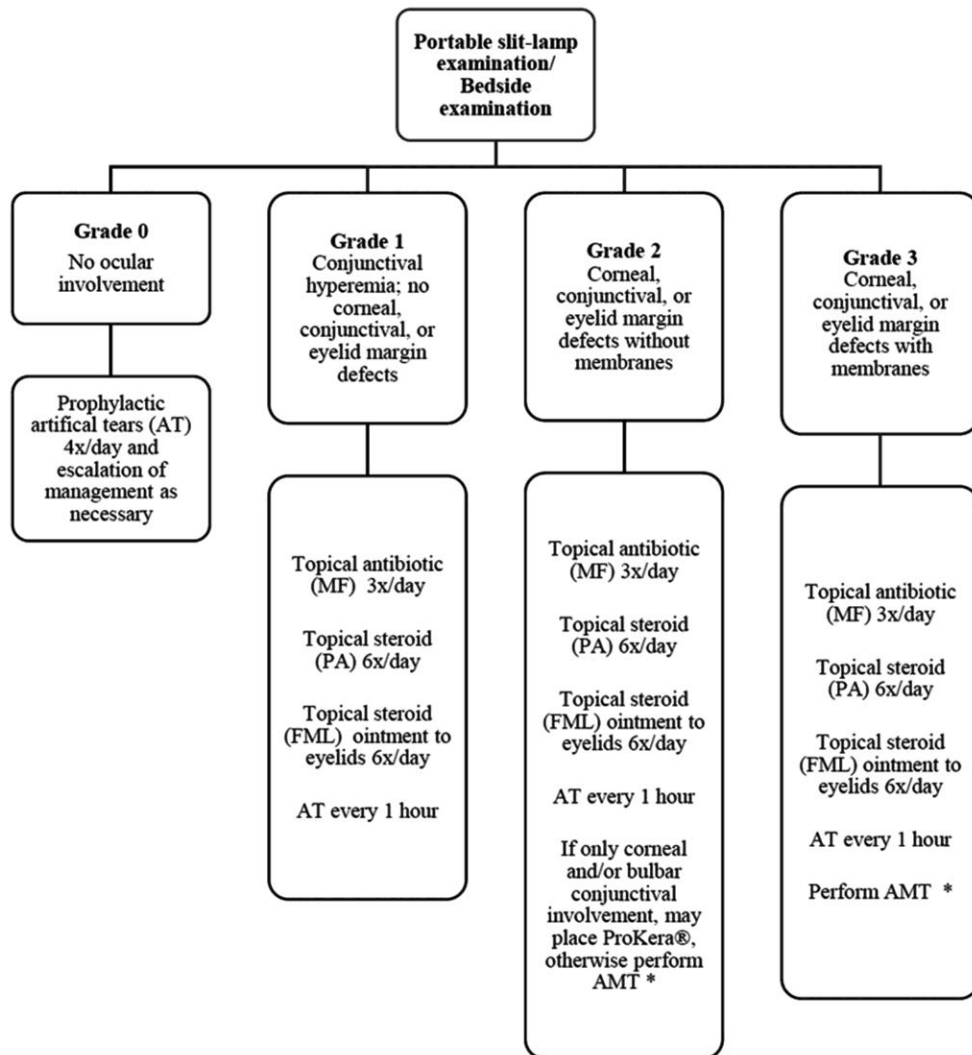

Supplement: Supplementary Figure 1 — Flow diagram outlining the protocol for management of ocular manifestations in acute SJS/TEN. MF [moxifloxacin 0.5%; PA [prednisolone acetate 1%; FML [fluorometholone 0.1%; AT [artificial tears; AMT [amniotic membrane transplantation. *Decision to perform AMT was based on feasibility (intubation status, cooperation, etc.). ProKera is acceptable only with limited bulbar conjunctival or corneal involvement or when AMT is not feasible. Reproduced with permission from Elsevier (23). [file Image_1.PDF]
